# Intermediate-term outcome after PSMA-PET guided high-dose radiotherapy of recurrent high-risk prostate cancer patients

**DOI:** 10.1186/s13014-017-0877-x

**Published:** 2017-08-23

**Authors:** Sebastian Zschaeck, Peter Wust, Marcus Beck, Waldemar Wlodarczyk, David Kaul, Julian Rogasch, Volker Budach, Christian Furth, Pirus Ghadjar

**Affiliations:** 1 Department of Radiation Oncology, Klinik für Radioonkologie und Strahlentherapie, Charité Universitätsmedizin Berlin, Augustenburger Platz 1, 13353 Berlin, Germany; 2 Department of Nuclear Medicine, Charité Universitätsmedizin Berlin, Berlin, Germany

**Keywords:** Psma-pet, Prostate-cancer, Salvage radiotherapy, Image based radiotherapy, PSA response

## Abstract

**Background:**

By the use of PSMA positron emission tomography (PET) detection of prostate cancer lesions with a high sensitivity and specificity combined with a favorable lesion to background contrast is feasible. Therefore, PSMA-PET is increasingly used for planning of radiotherapy treatment; however, any data on intermediate-term outcome is missing so far.

**Methods:**

Patients with high-risk or very high risk prostate cancer, referred for salvage radiotherapy (SRT, *n* = 22) between 2013 and 2015, underwent PSMA-PET prior to therapy. Irradiation was planned on PET data with boost to macroscopic tumors/metastases. Treatment related toxicity was measured using Common Terminology Criteria for Adverse Events (CTCAE, v4.0).

**Result:**

Findings in PSMA-PET led to treatment modifications in 77% of SRT patients compared to available CT information. One patient did not receive irradiation due to disseminated disease, the other patients received increased boost doses to macroscopic disease and/or inclusion of additional target volumes. Toxicity was low as only 2 patients reported toxicities > grade 1. With a Median follow-up time of 29 in patients that were not lost to follow-up, prolonged PSA responses below baseline were observed in the majority of patients (14 of 20). In hormone-naïve SRT patients (*n* = 11), radiotherapy led to prolonged PSA decrease in 8/11 patients, however with 3 of these 8 patients receiving repeated PSMA based irradiation of novel lesions during follow-up.

**Conclusion:**

PSMA-PET guided planning of radiotherapy led to change of treatment in the majority of patients. Treatment related toxicity was well tolerated and promising results regarding intermediate-term PSA decrease were observed.

**Trial registration:**

No trial registration was performed due to retrospective evaluation.

## Background

Both radiotherapy and radical prostatectomy are primary treatment modalities for localized prostate cancer. Cancer specific mortalities after these treatments is around 1% after 10 years for low and intermediate risk patients [1]. However, patient outcome is much less favorable for high-risk or very high-risk prostate cancer as these patients have a 10 year prostate specific mortality between 10 and 20% following definitive radiotherapy or surgery [2, 3]. This is probably due to the existence of unknown micro-metastatic disease prior to local treatment in a certain proportion of patients.

In case of biochemical failure (BF) after radical prostatectomy early salvage radiotherapy is the recommended treatment [4]. Postoperative treatment decision is commonly based on serum prostate specific antigen (PSA) values. One drawback of this approach is missing spatial information of recurrence, which would be highly relevant for the planning of a localized treatment like radiotherapy. Increased radiation dose was demonstrated to improve biochemical recurrence free survival after primary radiotherapy [5, 6] and was suggested to improve relapse-free survival in meta-analyses of retrospective data from salvage radiotherapy (SRT) patients [7, 8], a hypothesis which is currently evaluated by the phase 3 SAKK 09/10 trial [9]. However, dose intensification led to increased toxicity in primary treatment [5, 6] and was shown to negatively impact patients quality of life regarding urinary symptoms after SRT [9]. As a result, accurate detection of individual spread of disease or recurrence patterns would be highly warranted. Moreover, in selected oligometastatic patients radical treatment of identified metastatic lesions could further improve outcome.

Uncertainties about optimal treatment exist in a high risk post-operative setting with either high persisting PSA values after surgery or rapidly increasing PSA levels. If these patients benefit from SRT is not proven to date. In case of pre-SRT PSA values higher than 2 ng/ml the 4-year progression-free probability after SRT was only 19% whereas it is around 52% for patients with PSA values ≤2 ng/ml [10].

Recently there is a rapid increasing interest in positron emission tomography (PET) imaging using the tracer prostate specific membrane antigen (PSMA). PSMA is highly overexpressed at the surface of prostate cancer cells [11] and PSMA-PET was shown to be both, highly sensitive and highly specific, even in cases of low PSA values (<1 ng/mL) [12–15]. Thus, PSMA-PET may be especially relevant for radiotherapy treatment planning in a high-risk salvage setting. Due to high sensitivity and specificity of PSMA-PET even very small nodal metastases (<8 mm) are reliably detectable [16]. Additionally, due to the whole body approach even previously unexpected (distant) metastases can be localized. Thus, improvements in imaging may lead to an improvement in patient’s outcome. Therefore, we report on patients that received PSMA PET for high-risk SRT, i.e. patients with doubtful benefit of local standard treatment and focus und intermediate-term PSA control in these patients.

## Patients and methods

### Patient characteristics

Patients presenting at the department of radiation oncology for salvage radiotherapy after prostatectomy were stratified for their individual risk. High-risk patients as defined as pre-therapeutic PSA values above 20 ng/ml, Gleason score 8 or higher, persisting PSA values after radical prostatectomy (> 0.6 ng/ml), PSA values before SRT above 2 ng/ml or metastatic regional lymphnodes (diagnosed during prostatectomy), were referred for a pre-therapeutic PSMA PET/CT between 2013 and December 2015. If irradiation was still indicated after PSMA PET/CT imaging, patients were irradiated based on data of PSMA PET/CT. Originally 22 patients were scheduled for salvage radiotherapy (SRT) of the prostate bed. Median Age at the time of treatment was 65 years. Further details on the patient characteristics can be found in Table 1.

**Table 1:** Patient characteristics

Salvage Radiotherapy (SRT) *n* = 22	
PSA before surgery (ng/ml)	18.9 (3.14–115) Avg. (range)
Postoperative Gleason score	8 (6–10) Avg. (range)
PSA before SRT	6.1 (0.2–34.5) Avg. (range)
Months between surgery and SRT	29 (2–201) Avg. (range)
Concomitant androgen deprivation	10 of 22
Postoperative T stage	
T 2	7
T 3	14
T 4	1
Surgical margin status	
R 0	10
R 1	11
R 2	1
Postoperative PSA values	
PSA < 0.1 ng/ml	23
PSA > 0.1 ng/ml	9

### PET imaging

Images were acquired on a PET/CT device (Gemini TF 16 Astonish, Philips Medical Systems, Cleveland, OH, USA). Patients were examined in supine position from base of scull to the proximal femora 62 ± 35 min after intravenous injection of 113 ± 13.3 MBq of [^68^Ga]PSMA-HBED-CC (PSMA-11) (PSMA). In all patients, a diagnostic, contrast-enhanced CT was acquired and used for attenuation correction. For one patient, no information about injection time and injected dose was available, two patients received radiotherapy planning based on ^18^F–Cholin PET, and subsequently underwent PSMA-PET imaging.

The findings in PET imaging were categorized as follows: Local recurrence (T) included recurrence within the prostate bed and seminal vessels. Lymph node metastases within the regional drainage of the prostate was classified as N or as M1a if outside the regional drainage, further metastases (which were in all cases bone metastases) were scored as M1b.

### Radiotherapy treatment and clinical follow-up

Intensity-modulated radiotherapy (IMRT) was performed in all patients undergoing radiotherapy after PSMA-PET imaging. Almost all patients were treated with helical tomotherapy (Accuray, USA), two patients were treated with the Novalis™ radiosurgery system (Varian, USA; Brainlab, Heimstetten, Germany) and 2 patients received irradiation by linear accelerators (Varian, USA) using a volumetric modulated arc approach.

Radiotherapy prescription was as follows: Usually single fraction doses of 1.8 Gy were prescribed to a total dose of 66.6 Gy to prostate bed, including the base of seminal vesicles and the complete seminal vesicle in case of pT3b tumors. In the histologically described high-risk regions (with positive surgical margins or extracapsular spread) a simultaneous integrated boost (SIB) was applied to a cumulative total dose of 70.3 Gy. In case of PSMA-PET evidence of macroscopic local recurrence the SIB dose was increased to a maximal dose of 74 to 77.7 Gy. The lymphatic drainage was not irradiated unless if PET imaging revealed pelvic lymphnode metastasis. If the latter one was evident the lymphatic drainage was irradiated to a total dose of 54.0 Gy and macroscopic lymphnodes received a SIB to 66 Gy. Evident bony metastases were irradiated to a total dose of 42–66 Gy, depending on location and size. Androgen deprivation therapy (ADT) was prescribed according to the preference of the treating urologist.

Acute toxicities were scored at least every two weeks during treatment and at the end of therapy by the treating physician according to common terminology criteria for adverse events (CTCAE), version 4.0. Information about PSA values, toxicities and ADT was collected from the treating urologist and by patient interviews. PSA values were usually measured every 3 months following radiotherapy by the urologist, who was responsible for decision-making about initiation or continuation of ADT.

### Statistical calculations and software

Statistical analyses and plots were generated by GraphPad Prism 6 (Graphpad Software, La Jolla, CA, USA) and Inkscape 0.91 (www.inkscape.org).

## Results

### Modification of treatment according to findings in PSMA PET/CT

**Fig. 1:**
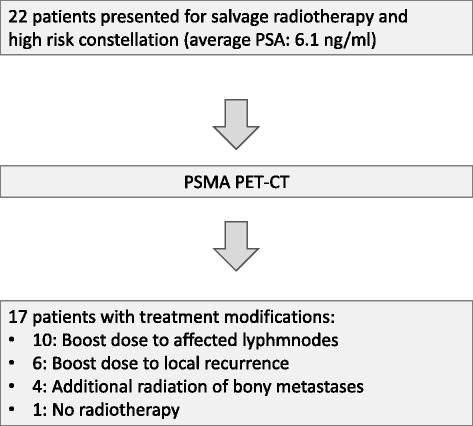
Treatment modifications after PSMA-PET in patients referred for salvage radiotherapy

In high-risk patients PSMA-PET led to treatment modifications in 77% patients referred for SRT. One patient presented with disseminated metastases and SRT was therefore omitted. Figure 1 depicts PSMA-PET based treatment modifications.

### Toxicity

Radiotherapy could be delivered as planned in all patients and was well tolerated (Table 2). Only 2 of 21 treated patients developed acute toxicities greater grade 1 according to CTCAE v4.0 (one case of grade 2 non-infective cystitis and one case of grade 2 diarrhea). During follow-up no late toxicities greater grade 1 were observed.

**Table 2:** Acute toxicities in irradiated patients

Toxicity	Grade 0	Grade 1	Grade 2
Salvage Radiotherapy (SRT) *n* = 21			
Proctitis	17	4	0
Diarrhea	14	6	1
Cystitis	13	7	1
Polakisuria	15	6	0
Fatigue	11	10	0
Other	19	2	0

### Follow-up

**Fig. 2:**
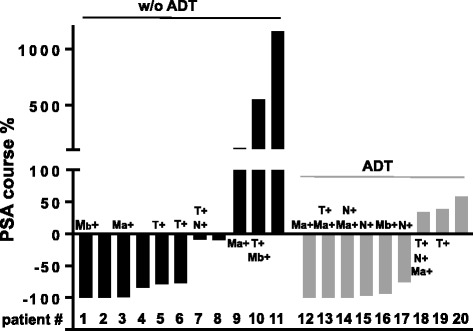
Percentual decrease/increase of PSA levels in individual patients from the time of salvage radiotherapy until last follow-up (average time: 30.2 months). Patients without (w/o) androgen deprivation therapy (ADT) in black and patients with ADT concomitant to radiotherapy in *grey*. Patient 11 initiated ADT during follow-up, patients 16 and 19 discontinued ADT longer than 6 months before measuring last PSA value. T+, N+, Ma + or Mb + indicates (irradiated) PSMA-PET findings of local, lymphnode or distant lymphnode (Ma+) or bony (Mb+) recurrences. Patients 3, 8 and 9 underwent re-irradiation of novel metastases during follow-up

One patient was lost to follow-up, median follow up time in the remaining irradiated 20 patients was 29 months (range: 12–49). Median PSA values at the time of last follow-up were 0.15 ng/ml (range: 0–8.45). 11 of these patients did not receive androgen deprivation therapy (ADT) prior or concomitant to SRT. During follow-up only one of these patients started ADT; however, three patients received PSMA-PET based irradiation of de novo lesions during follow-up. In this group of ADT naive patients the mean PSA value after an average follow-up time of 26 months (12–34) was 1.4 ng/ml (range: 0–5) with 8 out of 11 patients still presenting PSA values lower than prior to SRT. Figure 2 depicts the percentual PSA change between pre-SRT and last follow-up in all patients after SRT.

Out of 9 SRT patients with prior/concomitant ADT, 5 patients started ADT concomitantly to radiotherapy while 4 patients already presented a history of, sometimes long-term, ADT medication (average: 20 months, range: 3–58 months). Some of these patients received SRT with already rising PSA values during ADT (i.e. beginning of hormone refractory situation), which is probably the reason why some patients already presented increasing PSA values during intermediate-term follow-up (Fig. 2). 6 of the patients with ADT had substantial intermediate-term PSA responses. In three patients ADT could be or had to be omitted during follow-up.

Patients were grouped regarding clinical risk factors (Median initial PSA value and PSA value at the time of SRT, Gleason score up to 7 or higher, Median PSA Nadir value, postoperative T stage >3a and postoperative N+) and regarding PET findings (isolated local recurrence, no evidence of PSMA tracer uptake, any distant metastases or only lymphnode metastases N+ and/or Ma+). Median response rates for these different groups are summarized in Table 3. Differences in response were only observed in patients with pN+ and patients receiving salvage radiotherapy without any lesion in PSMA-PET (Median PSA response: −9% for PSMA negative patients versus −79% in case of irradiated PSMA lesions and +34% in case of pN+ versus −79% for pN0).

**Table 3:** Risk factors and their association with intermediate-term PSA response

Risk factor	-	+
Gleason score > 8	−18	−81
Median Initial PSA value	−76	−94
Median PSA Nadir	−78	−85
Median PSA before irradiation	−78	−86
Postoperative T stage >3a	−78	−85
Postoperative N+	−79	+34
PSMA-PET findings		
No detectable lesions	−79	−9
Isolated local recurrence	−89	−42
Distant metastases	−76	−89
Lymphnode metastases (N+/M1a)^a^	−76	−99

^a^Due to the low number of isolated regional recurrences (*n* = 2) these patients were combined with M1a patients for analyses

## Discussion

So far the impact of PSMA PET/CT on treatment outcome after radiotherapy is unknown. Here we report the first study with clinical meaningful follow-up longer than two years. We found first evidence that inclusion of PSMA PET/CT positive lesions for radiotherapy planning leads to favorable PSA responses in the majority of high-risk and very high-risk patients. Additionally, no high-grade acute toxicities were observed. Grade 2 toxicities were seen in 9.5% (2 of 21) of the irradiated patients only. The PET adapted radiotherapy planning can be regarded well tolerable, although PET imaging led to higher focal irradiation doses or inclusion of additional target volumes in 77% of patients (17 of 22 patients with SRT). Favorable intermediate-term PSA responses were observed in 8 of 11 hormone-naïve patients.

Currently data on PSMA-PET guided therapy is sparse and only a few studies assessed the impact of PSMA-PET on radiotherapy planning: According to previous retrospective studies PSMA-PET altered treatment decisions in 26% to 33% [17, 18] of primary treated patients and 42% to 61% of SRT patients [18, 19]. One study investigated treatment changes for a very heterogeneous group of patients, with the majority being SRT patients (67%), an reported an overall treatment adaptation rate of 46% [20]. Another recent publication which only analyzed SRT patients with PSA values below 1.0 ng/ml detected PSMA positive lesions in 54%, which potentially altered radiotherapy treatment of these patients [21]. Our data confirm the observations that radiotherapy is often altered in SRT after PSMA-PET imaging. An important difference is the rate of 77% in SRT which is higher than the average rate of the cited publications. This is probably due to a more restrictive use of PSMA-PET imaging in patients described here. Typically PSMA-PET was only applied in very high risk situations, in which the solely use of local therapies could be of doubtful benefit for patients.

The use of PSMA-PET is rapidly increasing in many countries, including Germany and Australia. This led to a recent expression of opinion from several experts in the field that the use of PSMA-PET has reached almost plague-like proportions [22]. National guidelines usually only propose PSMA-PET in case of recurrent disease as an optional method (German S3 guideline) or don’t even mention PSMA-PET (NCCN prostate cancer guidelines version 2.2017) [23, 24]. Due to the novel nature of PSMA-PET any long-term data, that include relevant endpoints like overall survival and prostate cancer specific survival, are missing so far. To our best knowledge, there is only limited data with short follow up, e.g. a mean follow-up of 8 months was recently published [19]. Our data has up to now the longest follow-up time and reveals interesting aspects in high-risk patients. Especially the finding that 8 of 11 ADT naïve patients receiving SRT have long-lasting PSA responses >2 years seems encouraging for the future use of PSMA based radiotherapy. Furthermore, in absence of this opportunity these very high risk patients would probably have been referred for ADT in the vast majority of cases. Androgen deprivation commonly fails after 2–3 years in case of macroscopic disease [25, 26]. Even in lower-risk patients with average PSA levels of 1.02 at the time of ADT initiation 13% of patients showed clinical progression within an average follow-up time of 2 years in a larger retrospective analysis [27]. Based on the relatively low case number it seems difficult to draw conlcusions on sub-groups that potentially benefit or do not benefit from PSMA-PET based radiotherapy. In our study patients with high-risk factors but without any evidence of PSMA lesions and patients with histological proven lyhmnode metastases (pN+) seemed to benefit less. However these findings should be validated in larger patient cohorts.

For the here described high-risk constellation SRT candidates clinical evidence and guidelines are sparse. Based on a retrospective analysis from Trock and colleagues common criteria to determine if patients benefit from SRT are: SRT within 2 years of BCR and no persisting PSA levels after surgery [28], however another study proved positive effects of SRT even with longer time intervals, at least for T3 tumors [29]. While the study from Trock did not find a correlation between pre SRT PSA values and outcome after radiotherapy, although patients with pre-SRT PSA values up to 57 ng/ml were included, other studies found a positive effect of SRT mainly in patients with PSA values below 1 ng/ml [30]. The multi-istitutional analysis from Tendulkar and colleagues supports the notion that optimal SRT should be delivered as early as possible with low PSA values [31]. The ideal treatment for patients with higher PSA values or a longer time interval between surgery and intervention is therefore unclear.

In case of oligometastastatic prostate cancer radical local therapy including radiotherapy seem to be associated with favourable patient outcome, although any prospective data on this important issue is lacking [32, 33].

## Conclusions

Our data, although limited by its retrospective nature and small sample size, support the role of radiotherapy in combination with state of the art PSMA-PET imaging for individually tailored treatment. The low rate of toxicities and the high rate of durable PSA response are encouraging and merit further prospective evaluation. We are well aware that due to restrictions of imaging resolution, PET based radiotherapy mostly only hits the tip of the iceberg in patients presenting a tumor stage where microscopic spread beyond PET lesions seem to be the rule and not the exception. Therefore we decided against the use of classical criteria for biochemical recurrence, since probably almost all patients described here will relapse. Instead the relative increase/decrease of PSA levels compared to the level at initiation of radiotherapy was applied. As PSA levels are not only highly relevant for the psychological wellbeing of prostate cancer patients, but also for decision making regarding ADT or therapy intensification, an intermediate-term gain of PSA levels below baseline seems to be an appropriate endpoint in this setting. In this regard our findings indicate the safety and effectiveness of PSMA based radiotherapy in the described high-risk patients, having in mind that prospective data for optimal treatment for these patients is currently missing. However these patients should be informed that the radiotherapeutic approach is probably only able to delay ADT as during follow-up arising of novel lesions seems to be frequent, which however often can be temporary treated locally, too (3 of 11 cases received PSMA-PET based irradiation during follow-up).

## Abbreviations

ADT: Androgen deprivation therapy
BF: Biochemical failure
CT: Computed tomography
CTCAE: Common terminology criteria for adverse events
Gy: Gray
IMRT: Intensity-modulated radiotherapy
PET: Positron emission tomography
PSA: Prostate specific antigen
PSMA: Prostate specific membrane antigen
SIB: Simultaneous integrated boost
SRT: Salvage radiotherapy
